# On the Treatment of Internal Strangulation of the Intestines by Strychnine

**Published:** 1849-07

**Authors:** 


*On the Treatment of Internal Strangulation of the Intestines by Strychnine.*—Dr. Homolle relates three cases in which he found very minute doses of strychnine (one millegramme) repeated every hour completely efficacious, after various other means had been tried in vain in relieving urgent symptoms of internal strangulation, produced in two of the cases by the return of the hernia *en masse*, and in the other by violent vomiting. It was the knowledge he had of the favorable influence which strychnine exerts upon the peristaltic action of the bowels, in constipation from cerebral paralysis, hypochondriasm, etc., that induced him to employ it in the present cases. The borborygmi, the painless sense of vermicular movement, the rapid cessation of the pain and vomiting, and the expulsion, first of gas and then of feces, which resulted from its employment, confirmed the anticipation he had entertained. M. Amussat, who employs the conjoined force of two or more persons for the reduction of hernia, believes that in this way nine-tenths may be reduced; but the taxis is, in point of fact, but the substitution of an external constricting force for the insufficient contraction of the walls of the intestine. The alkaloid by increasing defective, or by correcting irregular peristaltic action, diminishes the volume of the intestine, and expels the retained fecal matters, or removes invaginations. It is to be recollected that the obstacle in strangulation of the intestine is hardly ever materially insurmountable. So that this means may sometimes associated with the taxis, even in strangulated hernia, prevent the necessity of resorting to an operation.—*L'Union Médicale.—British and Foreign Medico-Chirurgical Review.*

